# PDGF signaling in cartilage regeneration: molecular mechanisms and cellular effects

**DOI:** 10.3389/fcell.2026.1778703

**Published:** 2026-02-25

**Authors:** Qiang-qiang Zhao, Feihong Che, Weiwen Liu, Chengyu Hou, Lijuan Yu, Yonghui Hou, Liangliang Xu

**Affiliations:** 1 Lingnan Medical Research Center, The First Affiliated Hospital of Guangzhou University of Chinese Medicine, Guangzhou, China; 2 Lingnan Medical Research Center, Guangzhou University of Chinese Medicine, Guangzhou, China; 3 School of Basic Medical Science, Guangzhou University of Chinese Medicine, Guangzhou, China; 4 Department of Orthopedic Surgery, Guangdong Provincial Hospital of Chinese Medicine, The Second Affiliated Hospital of Guangzhou University of Chinese Medicine, Guangzhou, China

**Keywords:** cartilage, chondrocyte, mesenchymal stem cell, osteoarthritis, PDGF

## Abstract

Cartilage damage has long been a challenge in regenerative medicine research, due to the avascular nature of cartilage tissue and its intrinsically limited reparative capacity. In recent years, more and more studies have found that the regulation of signaling factors at the temporal, spatial, and dose levels is one of the key factors restricting the effective regeneration of cartilage. As a classic growth factor, platelet-derived growth factor (PDGF) plays an important regulatory role in cell recruitment, proliferation, and tissue repair, and has gradually become the focus of cartilage regeneration research. Evidence has shown that PDGF exhibits significant context-dependent regulatory characteristics in different microenvironments. This review systematically reviews the structural characteristics and signal transduction pathways of different PDGF isoforms and their receptors (PDGFR-α and PDGFR-β), focusing on their biological roles at the cellular level, including the regulation of mesenchymal stem cell migration and expansion, the impact on chondrocyte survival and metabolic activity, and their bidirectional regulatory effects in the synovium-cartilage axis and osteoarthritis-related microenvironment.

## 1 Introduction

Articular cartilage is a highly specialized connective tissue characterized by a lack of vascular, nerve, and lymphatic distribution, as well as very low cell density, with chondrocytes typically making up less than 5% of the total tissue volume (Hunziker, 2002; Huang and Cai, 2025). These structural features are essential for maintaining long-term mechanical stability and load-bearing function of joints, but they also put articular cartilage in a special microenvironment with limited innate repair ability. After injury, due to insufficient vascular supply, the effective recruitment of inflammatory cells, progenitor cells, and stem cells is limited, resulting in difficulty in fully releasing repair-related signaling molecules, which ultimately leads to a decrease in repair efficiency. Therefore, the area of articular cartilage defect is often filled with fibrocartilage, making it difficult to form hyaline cartilage with normal structure and function, which eventually leads to joint degeneration and functional decline (Lories and Luyten, 2010). This process is considered an important pathological basis for the occurrence and development of osteoarthritis.

From the histological level, the main reasons for the difficulty of healing mature articular cartilage are as follows: First, the number of chondrocytes is limited and highly differentiated, and the migration and proliferation ability is significantly limited (Zhou et al., 2023; Correa and Lietman, 2017); Second, although the densely cross-linked extracellular matrix (ECM) confers good mechanical properties to cartilage, it also constitutes the main physical barrier for exogenous repair cell infiltration and its stable integration (Heinegard and Saxne, 2011). In the context of osteoarthritis, these structural limitations are further aggravated, with a progressive phenotypic instability of chondrocytes, accompanied by disruption of the local ECM microstructure, thinning of the cartilage layer, or even complete absence, ultimately leading to exposure of subchondral bone (Dieppe and Lohmander, 2005).

At the molecular level, cartilage damage rapidly promotes the transformation of the local microenvironment into a catabolic state. Multiple pro-inflammatory cytokines, such as interleukin-1β (IL-1β) and tumor necrosis factor-α (TNF-α), are consistently expressed at high levels, which in turn activate key signaling pathways such as NF-κB and MAPK, inducing the expression of a variety of matrix-degrading enzymes, including matrix metalloproteinases (MMPs) and members of the ADAMTS family (Wang Y. et al., 2025; Xu et al., 2009). This series of changes accelerates the degradation of type II collagen and proteoglycans, continuously disrupting the structural integrity of the cartilage matrix (Sokolove and Lepus, 2013). At the same time, this chronic, low-grade but persistent inflammatory state not only hinders the effective reconstruction of the stroma but also inhibits the activation of pro-regenerative signaling pathways, further limiting endogenous cartilage repair (Goldring and Otero, 2011; Li et al., 2011). In the chronic progression of osteoarthritis, the catabolic signals dominate for a long time, gradually forming a stable low-grade inflammatory microenvironment, which not only leads to continuous depletion of the extracellular matrix of chondroblast but also further exacerbates the disruption of cartilage homeostasis by inhibiting synthesis and regeneration-related signaling pathways, including those mediated by TGF-β and IGF-1 (Zhao et al., 2014; Mourkioti and Rosenthal, 2005).

Among the many growth factors, PDGF, which is related to cartilage regeneration, has not been paid attention to for a long time, and its potential biological value has not been systematically explored in this field. PDGF is often considered a mitotic or fibrotic factor with a focus on angiogenesis, bone repair, and soft tissue healing. In contrast, PDGF is often considered to lack the ability to directly induce cartilage differentiation (Indrawattana et al., 2004), which limits its application in therapeutic strategies centered on cartilage regeneration to some extent. However, the accumulating research evidence in recent years is gradually revising this understanding. Studies have shown that PDGF, especially the PDGF-BB isomer, plays a key and irreplaceable regulatory role in the early stages of cartilage regeneration (Liebesny et al., 2016). In addition, it has been found that PDGF can activate multiple intracellular signaling pathways, including PI3K–Akt, thereby alleviating inflammation-related chondrocyte apoptosis and matrix degradation to a certain extent (Omata et al., 2023), and helping to improve the local inflammatory microenvironment that is not conducive to repair.

PDGF signaling should not be simply regarded as antagonistic to the cartilage differentiation pathway, but rather as an upstream regulator in the cartilage regeneration process, which provides the necessary cellular and microenvironmental basis for subsequent cartilage formation mediated by classical differentiation signals such as TGF-β and BMPs by promoting the recruitment and expansion of early repair cells (Buket Basmanav et al., 2008). Therefore, classifying PDGF only as a non-specific proliferative factor may underestimate its integrated role in the regulatory network of cartilage repair and regeneration.

## 2 Molecular architecture and functional basis of the PDGF–PDGFR axis

To understand the diverse functions of platelet-derived growth factor (PDGF) signaling, the molecular structure of the PDGF-PDGFR axis must first be clarified.

### 2.1 PDGF isoforms and structural features

**FIGURE 1:**
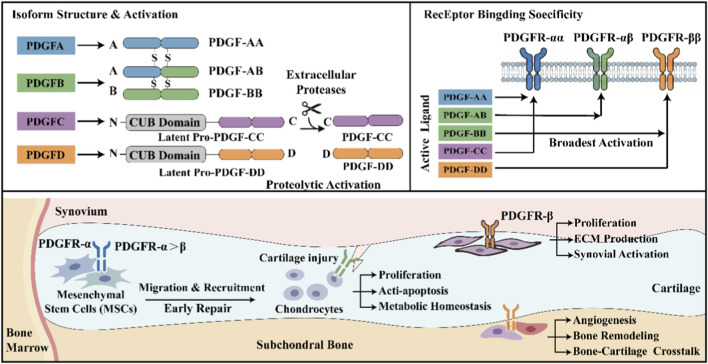
PDGF isotypes, receptor specificity, and cellular function during cartilage repair. This diagram details the proteolytic activation process of PDGF isoforms and their differential binding to PDGFR dimers, while indicating the broad specificity of PDGF-BB. It further illustrates how cell-specific PDGFR expression determines different functional responses in MSCs, chondrocytes, and synovial tissues, thereby coordinating the cartilage repair process.

The PDGF family consists of a group of dimeric growth factors that were originally found in α particles of platelets. With the development of molecular biology, it has now been confirmed that the PDGF family consists of four homologous polypeptide chains encoded by the PDGFA, PDGFB, PDGFC, and PDGFD genes (Figure 1). These polypeptide chains undergo dimerization after translation through the formation of disulfide bonds, resulting in biologically active PDGF ligands (Fredriksson et al., 2004).

To date, five PDGF isomers have been identified, including PDGF-AA, PDGF-AB, PDGF-BB, PDGF-CC, and PDGF-DD. Among them, PDGF-AA, PDGF-AB, and PDGF-BB are considered classical PDGF isomers, while PDGF-CC and PDGF-DD have different precursor structures (Li and Eriksson, 2003; Fredriksson et al., 2004). Specifically, PDGF-C and PDGF-D are secreted as latent precursor proteins containing N-terminal CUBs (C1r/C1s, Egf, Bmp1) domains. Hydrolytic removal of this N-terminal domain by extracellular proteases exposes the C-terminal growth factor domain, allowing for receptor binding and activation. This protease-dependent activation mechanism confers higher spatial and temporal specificity to PDGF-C and PDGF-D, making their biological activity highly dependent on the local proteolytic microenvironment (Li and Eriksson, 2003; Fredriksson et al., 2004).

From a structural and functional perspective, different PDGF isoforms differ significantly in terms of receptor binding preferences and signaling potency. PDGF-AA binds primarily to PDGFR-αα homomers, while PDGF-AB can activate PDGFR-αα and PDGFR-αβ isomers (Roskoski, 2018). Notably, PDGF-BB is the only isoform capable of efficiently activating all three receptor configurations (PDGFR-αα, PDGFR-αβ, and PDGFR-ββ) with high affinity. This broad pattern of receptor activation explains the strong and relatively consistent biological effects of PDGF-BB observed in various tissue repair and regeneration scenarios (Heldin and Lennartsson, 2013).

In the context of cartilage regeneration, the structural properties of PDGF-BB are particularly important. Its homomeric conformation provides greater stability at the receptor binding interface, leading to more efficient and durable receptor phosphorylation and downstream signal transduction (Peng et al., 2020; Povysil et al., 2025). These molecular features provide a structural basis for the dominant role of platelet-derived growth factor-BB (PDGF-BB) in promoting the proliferation, migration, and regulation of extracellular matrix metabolism in chondrocytes and mesenchymal stem cells (MSCs) (Hye-Ryong Shim et al., 2010).

### 2.2 Cellular distribution of PDGFR-α and PDGFR-β

The biological effects of PDGF are primarily mediated by PDGFR-α and PDGFR-β. Although the two are structurally highly homologous, there are significant differences in tissue distribution and functional focus, and this heterogeneity at the receptor level constitutes an important basis for the context-dependent regulatory effects of PDGF signaling during cartilage repair and regeneration (Heldin and Lennartsson, 2013).

In MSCs, PDGFR-α and PDGFR-β are expressed, with PDGFR-α usually dominant (Dhahri et al., 2016; Camorani et al., 2017). Studies have shown that PDGFR-α-positive MSC subsets have greater migratory capacity and clonal formation potential, and are more sensitive to PDGF-AA and PDGF-CC. Activation of PDGF signaling promotes the mobilization of these cells from storage sites such as bone marrow, synovium, or subchondral bone and recruitment to the injured area, providing a key source of cells for the early stages of cartilage repair (Morikawa et al., 2009). PDGFRs expression is low in mature chondrocytes under physiological conditions, but is significantly upregulated in the presence of injury, inflammation, or degenerative changes, especially in PDGFR-β (Schmidt et al., 2006). Functional studies have shown that PDGF mainly regulates chondrocyte proliferation, anti-apoptosis, and metabolic homeostasis, rather than directly inducing terminal differentiation, and is therefore more likely to play a supportive role in the early to mid-stage repair (Zhao et al., 2016).

Synovial cells are also important targets of PDGF signaling, especially in the context of osteoarthritis. The high expression of PDGFR-β in fibroblastic synovial cells makes it highly sensitive to PDGF, which promotes its proliferation and matrix synthesis, but may also enhance synovial activation in the inflammatory microenvironment and indirectly affect cartilage homeostasis through the synovium-cartilage axis, reflecting its “double-edged sword” characteristics (Charbonneau et al., 2016).

In addition, PDGFR-β is also highly expressed in osteogenic-associated cells and vascular-associated cells in the subchondral bone region (Wang et al., 2023), where PDGF signaling is involved in the regulation of angiogenesis, bone remodeling, and signal communication at the bone-cartilage interface, which are considered to be key factors affecting the quality of cartilage repair and long-term structural stability, further highlighting the multi-level and context-dependent role of PDGF in the joint microenvironment (Crisan et al., 2008).

## 3 Roles of PDGF in cartilage regeneration

**FIGURE 2:**
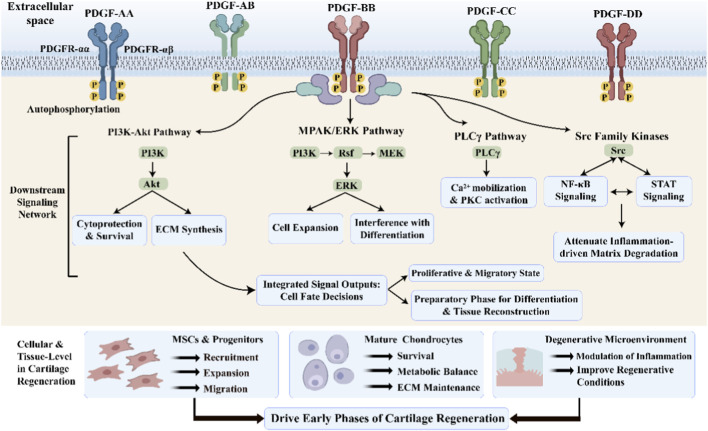
The PDGF-PDGFR signaling network in cartilage regeneration. This schematic illustrates how PDGF ligand binding activates PDGFRs, initiating downstream cascades primarily including PI3K-Akt, MAPK/ERK, and Src family kinases. Integrated signals from these pathways coordinate responses in MSCs and mature chondrocytes, while modulating the degenerative microenvironment to drive the early phases of cartilage regeneration.

In addition to the previously described PDGF ligands and their isoforms, the expression of PDGFR-α and PDGFR-β in different cell populations of joint tissue also showed significant spatiotemporal heterogeneity. Selective activation of specific ligand-receptor combinations triggers downstream signals adapted to the microenvironment and regulates cellular fate and tissue responses through hierarchical and cross-pathway integration (Heldin and Lennartsson, 2013). Therefore, hierarchical elucidation of the PDGF–PDGFR system from the ligand-receptor-signaling network axis is of key significance to understand its regulatory role in cartilage regeneration (shown in Figure 2).

### 3.1 The PDGF–PDGFR signaling axis

When PDGF ligands bind to their receptors, PDGFRs dimerize and subsequently rapidly autophosphorylate on specific tyrosine residues in the intracellular domain. These phosphorylation sites provide a binding platform for adaptor and effector proteins carrying Src homology domain 2 (SH2) or phosphotyrosine binding (PTB) domains, thereby initiating complex downstream signaling (Lemmon and Schlessinger, 2010). Studies have shown that PI3K–Akt, MAPK/ERK, PLCγ, and Src family kinases form the core signaling modules of the PDGF system (Chung et al., 1994; Valius and Kazlauskas, 1993).

PDGF signaling can interact with inflammatory and stress pathways, such as NF-κB and STAT, through Src family kinases. In the degenerative joint microenvironment, these signals help mitigate inflammation-driven matrix degradation and indirectly improve the environment for cartilage regeneration (Brentano et al., 2007). It is important to note that the PDGF downstream signaling pathway does not exist in isolation, but is a dynamic and feedback-regulated network (Chung et al., 1994). Bidirectional regulation between the PI3K–Akt pathway and the MAPK pathway modulates signal output, ultimately determining cell proliferation and migration. These regulatory properties explain the differential effects of PDGF on cartilage regeneration at different stages.

The molecular framework of the PDGF–PDGFR axis can be established through systematic analysis of PDGF isosome properties, receptor distribution, and downstream signaling networks (Heldin and Lennartsson, 2013). In this context, PDGF plays a central regulatory role in the early stages of cartilage regeneration, influencing the regeneration process by coordinating the responses of different cell types and tissue sites. Next, the effects of PDGF on cells will be analyzed from three complementary perspectives MSCs, mature chondrocytes, and the degenerative/osteoarthritis microenvironment) to provide a mechanistic basis for translational applications and regenerative strategies (Fortier et al., 2011).

### 3.2 Chemotactic and proliferative effects of PDGF on mesenchymal stem cells

Efficient recruitment (homing) of MSCs is a critical step in cartilage repair. PDGF-BB has significant chemotaxis effects on a variety of mesenchymal mass lineage cells, mainly through the activation of PDGFR-mediated migration signaling and cytoskeletal reorganization (Liebesny et al., 2016). In the inflammatory or injury microenvironment, PDGF-BB acts as an endogenous “guide signal” that drives MSC migration to high-concentration PDGF-BB regions via the Src–Akt signaling cascade; Blocking Src or Akt signals significantly reduces the homing efficiency of MSCs, indicating that the PDGF-BB/PDGFR–Src–Akt axis has a key function in the chemotaxis process (Zimmer et al., 2009). In addition, PDGF coordinates PI3K/Akt and PLCγ pathways to regulate small GTPases and focused attachment site dynamics, promoting pseudopodia formation and cell polarity establishment. MEK/ERK and Src modules are involved in signal amplification and integration. In areas of cartilage defects, PDGF can bind to the extracellular matrix to form a concentration gradient that provides continuous directional guidance for MSCs, which is often designed as a mechanism for early release or surface fixation, primarily for recruiting endogenous MSCs rather than long-term high-dose delivery (Caplan, 2007). However, the ultimate fate of MSCs still depends on subsequent microenvironmental signals. In addition to chemotactics, PDGF is a recognized mitogen of MSCs. Activation of PDGFR tyrosine kinase promotes the MAPK–ERK pathway and related cell cycle programs, driving MSC proliferation, while PI3K–Akt improves metabolic fitness and enhances anti-apoptotic capacity, allowing MSCs to survive under hypoxia, nutrient-deprived, or inflammatory conditions (Kratchmarova et al., 2005). This combined effect of promoting proliferation and survival makes PDGF particularly suitable in the early stages of cartilage repair (Fortier et al., 2011).

However, the strong proliferative effect of PDGF also poses challenges. Proliferation and terminal differentiation are often mutually regulated: MSCs progressing to the cartilage lineage need to change from a proliferative state to a state characterized by matrix synthesis and phenotypic stabilization (Mueller and Tuan, 2008). Persistent or excessive PDGF signaling may maintain high MSC proliferation, thereby interfering with the establishment of chondrogenic transcriptional networks. This is consistent with the pattern observed in multiple differentiation systems, where long-term ERK high activation inhibits terminal differentiation and is one of the key stage-dependent issues for PDGF application in cartilage regeneration (Shi et al., 2009).

### 3.3 Stage-dependent regulation of chondrogenesis by PDGF

In the early stages of cartilage regeneration, including MSC recruitment, adhesion, and proliferation, short-term exposure to PDGF can maintain cell viability and migration while enhancing responsive plasticity to subsequent induced signals (Murphy et al., 2013). In contrast, the middle and late stages are characterized by enhancement of chondrogenesis gene programmes and significant extracellular matrix deposition, and sustained PDGF stimulation is often associated with impaired terminal chondrogenesis, manifested by insufficient expression of COL2A1 and ACAN, decreased phenotypic stability, and even a shift to a fibrochondral phenotype (Bobick and Kulyk, 2004). Studies have shown that PDGFRα signaling plays a regulatory role in the bifurcation of cartilage-fibrous lineages, and its dynamic overlap with Sox9 expression in embryo-specific progenitor cell populations suggests that PDGFRα does not simply promote cartilage differentiation, but plays a “navigation” role at key nodes of cell fate selection (Tallquist and Soriano, 2003). Therefore, the biological function of PDGF is more biased towards regulating developmental time and cellular potential rather than directly driving mature cartilage formation. Selecting the appropriate time to remove PDGF and using differentiation signals such as TGF-β or BMPs, biomechanical stimulation, and ECM signals that mimic cartilage can introduce chondrocytes into the mature differentiation stage. In the absence of this temporal regulation, MSCs may remain proliferative for a long time, delaying or inhibiting their cartilage differentiation (Huebsch et al., 2015).

In addition, the differentiation process of MSCs relies on the synergy of multiple signaling pathways, including TGF-β, Wnt, MAPK, and Hippo/YAP, among others, where PDGF plays an upstream regulatory role rather than a direct directive signaling role. Studies have shown that the ERK/Sox9 axis is a key regulatory node, indicating that PDGF mainly affects Sox9 indirectly by regulating ERK activity, thereby affecting the cartilage differentiation process (Lutolf and Hubbell, 2005). Overall, the core value of PDGF lies in expanding the number of MSCs and maintaining their functional state, laying the foundation for cartilage differentiation. Therefore, precise time control and multifactorial coordination are key to PDGF’s cartilage regeneration.

### 3.4 Effects of PDGF on chondrocytes and joint microenvironment

Mature chondrocytes are the main effector cells of hyaline chondrocyte extracellular matrix (ECM) production, which are directly responsible for the synthesis and maintenance of the matrix. The biological effects of PDGF on chondrocytes can be summarized as promoting proliferation, anti-apoptosis, and having anti-inflammatory or chondroprotective effects under specific conditions. Importantly, these effects are not uniform, but clearly depend on conditions such as dose, exposure time, and microenvironment (Chen et al., 2025).

#### 3.4.1 PDGF-mediated promotion of chondrocyte proliferation and survival

PDGF notably enhances chondrocyte proliferation during initial repair phases and *in vitro* expansion. *In vivo*, mature chondrocytes have limited proliferation capacity, and repair after injury mainly relies on limited expansion and migration of lesional marginal cells. PDGF-BB has been shown to enhance chondrocyte proliferation by activating the ERK1/2 pathway, which is supported by evidence in both platelet-rich plasma (PRP) and chondrocyte culture systems (Depypere et al., 2020; Cucchiarini et al., 2005). In addition, this proliferation is also applicable to cell therapies such as autologous chondrocyte engraftment (ACI) and stroma-assisted ACI (MACI), in which *in vitro* expansion is inevitable but is often accompanied by cell dedifferentiation (Xiao et al., 2014). Studies have shown that PDGF not only accelerates the cell cycle but also affects cell proliferation by regulating the cytoskeleton and cell-matrix adhesion by affecting dedifferentiation trajectories (Wang Z. et al., 2025). In the microenvironment of cartilage damage and osteoarthritis (OA), apoptosis and matrix degradation form a cycle, which is the core of cartilage degeneration. The anti-apoptotic effects of PDGF-BB are mainly achieved through the PI3K/Akt and ERK pathways, which have been reported in a variety of cell types (Park-Min, 2019).

In OA-related models, recombinant PDGF-BB attenuates chondrocyte apoptosis by downregulating caspase-3 signaling, while reducing matrix loss and increasing COL2 expression (Zhu et al., 2021; Schneider et al., 2011). These results suggest that PDGF may have chondroprotective effects under certain conditions. However, the results vary across models, doses, and administration strategies, and the final effect depends on the overall state of the joint microenvironment.

#### 3.4.2 Dual regulation of ECM metabolism and inflammatory responses by PDGF

PDGF is environmentally dependent on the regulation of chondrocellular extracellular matrix (ECM) synthesis. In inflammatory or stressful settings, PDGF-BB is often observed to enhance the expression of cartilage-specific markers such as proteoglycan and type II collagen while mitigating matrix degradation. These effects are often accompanied by inhibition of NF-κB signaling and activation of the Src and PI3K/Akt pathways, thereby partially counteracting IL-1β-induced apoptosis and ECM degradation. The results suggest that the anti-inflammatory and anti-apoptotic effects of PDGF may be closely related to short-term ECM protection (Omata et al., 2023).

However, sustained platelet-derived growth factor (PDGF) signaling disrupts the phenotype of chondrocytes, suggesting that the synthesis of extracellular matrix (ECM) supported by PDGF is environment-specific and time-constrained (Bobick and Kulyk, 2004). In the complex inflammatory milieu of injured or osteoarthritic joints, PDGF displays a dual profile characterized by the coexistence of protective and amplifying effects. On the one hand, PDGF-BB can suppress NF-κB activation and mitigate apoptosis and catabolic signaling in chondrocytes exposed to IL-1β. On the other hand, as a potent receptor tyrosine kinase ligand, PDGF may engage in crosstalk with stress- and inflammation-related pathways such as JAK/STAT and p38, and exert indirect effects via non-chondrocytic cell populations, including synovial fibroblast-like cells, thereby potentially amplifying inflammatory and fibrotic responses (Brentano et al., 2007).

Given that OA is increasingly recognized as a “whole-joint” disease involving cartilage, synovium, subchondral bone, and neurovascular components (Lories and Luyten, 2010), and that PDGF receptors are broadly expressed across these tissues (Morikawa et al., 2009; Crisan et al., 2008), the net biological outcome of PDGF signaling cannot be readily extrapolated from chondrocyte-centric observations alone. Accordingly, PDGF should neither be simplistically classified as a unidirectional chondroprotective factor nor broadly labeled as a pro-degenerative signal; rather, its effects in inflammatory environments are highly contingent on target cell populations, microenvironmental status, and the temporal design of intervention strategies.

#### 3.4.3 Convergence of reparative signaling and pathological remodeling

During OA progression and cartilage repair, the synovium–cartilage axis and the subchondral bone–vascular unit together form a tightly coupled regulatory network (Sanchez-Lopez et al., 2022; Tuckermann and Adams, 2021). Synovial-derived cytokines and proteases accelerate cartilage degradation, while cartilage injury-associated molecular patterns can, in turn, activate synovial inflammation, sustaining a state of chronic joint stress. The detection of PDGF and its receptors in inflamed synovial tissue, together with the reported expression of specific PDGF isoforms in synovium, suggests that PDGF signaling may participate in synovial pathological processes (Appleton et al., 2007).

Therefore, strategies aimed at enhancing PDGF signaling through intra-articular injection or biomaterial-based pathways are unlikely to act solely on chondrocytes. Short-term or moderate PDGF exposure may contribute to the initiation of repair, while long-term or diffuse exposure may trigger synovial activation, fibrous remodeling, or pain-related structural changes that ultimately affect tissue repair (Su et al., 2020). Emerging evidence suggests that endothelial cell-associated PDGF signaling may exacerbate osteoarthritis-related changes by promoting vascularization and coupled bone remodeling. This view provides a coherent explanation for the seemingly contradictory conclusions reported in PDGF studies: PDGF may appear beneficial when evaluated only in terms of chondrocyte anti-apoptotic or anti-inflammatory effects; However, if synovial fibrosis, vascular invasion, and immune cell recruitment are included in the analytical framework, PDGF may be one of the factors leading to pathological remodeling (Hamilton et al., 2016).

Therefore, these results are not exclusions, but reflect the multi-target, multi-pathway properties of PDGF at different levels. Therefore, the final biological outcome of the PDGF intervention depends on the combined influence of multiple factors.

## 4 Discussion

This article systematically reviews the biological role of PDGF signaling axis in cartilage regeneration and its regulatory characteristics. Based on the existing basic and translational research evidence, PDGF should not be defined as a factor that induces cartilage terminal differentiation, but should be positioned as a key regulatory molecule with early repair regulation as its core function. Its main biological significance is to promote the recruitment, expansion, and survival of mesenchymal stem cells (MSCs) and maintain chondrocyte function under inflammatory or stress conditions, thereby laying the foundation for subsequent tissue reconstruction at the level of cell composition and microenvironment.

From a mechanism of action perspective, PDGF does not directly drive phenotypic maturation of cartilage, but rather determines whether the repair process has the ability to “initiate” and “sustain” by regulating cell number, viability, and homeostasis of the microenvironment. This understanding helps explain the differences in the effects of PDGF across studies, not due to uncertainty in PDGF function itself, but more in the timing of its application, dose control, and synergy with other signaling pathways. Notably, the application of PDGF alone, continuously, or non-specifically often makes it difficult to achieve stable hyaline cartilage regeneration and may even induce fibrotic tendencies or joint homeostasis disorders. Therefore, PDGF should not be used alone as a single factor throughout the regeneration process, but should be incorporated into a systematic, designed regulatory framework.

Basic research provides a clear path for the clinical translation of platelet-derived growth factor (PDGF). First, at the temporal regulation level, PDGF is more suitable for the early “initiation and expansion stage” of repair, while the differentiation and maturation signals represented by TGF-β, BMP, etc., should be gradually introduced in the later stage to achieve a smooth transition from “cell preparation” to “tissue construction”. Secondly, at the spatial and microenvironmental regulatory level, the combination of PDGF with controlled-release biomaterial and tissue-engineered scaffolds can help achieve its local and precise effects, thereby improving safety and predictability. Finally, in terms of joint regulatory strategies, PDGF should be used as a key node in the regenerative signaling network, cooperating with various growth factors to construct a regulatory system close to the physiological repair process.

At the specific translational level, platelet derivatives provide a practical model for the engineering application of PDGF, and their multifactorial synergistic properties are closer to the physiological repair microenvironment, so they have high clinical acceptability. In the future, it is expected that PDGF’s targeting and stability in cartilage regeneration will be further improved by finely regulating its content, release kinetics, and proportional relationship with other factors.

In general, PDGF still has important development potential in the field of cartilage regeneration, but its real value does not lie in the “single factor driven”, but in the organic integration with a controllable and predictable systematic regulatory framework to become a regulatory hub for regeneration signals. With advances in multidisciplinary and engineering techniques, PDGF is expected to gradually transform from a long-controversial growth factor to a finely regulated and convertible regenerative medicine tool molecule.

## References

[B1] Appleton C. T. G. Pitelka V. Henry J. Beier F. (2007). Global analyses of gene expression in early experimental osteoarthritis. Arthritis and Rheumatism 56 (6), 1854–1868. 10.1002/art.22711 17530714

[B2] Bobick B. E. Kulyk W. M. (2004). The MEK-ERK signaling pathway is a negative regulator of cartilage-specific gene expression in embryonic limb mesenchyme. J. Biol. Chem. 279 (6), 4588–4595. 10.1074/jbc.M309805200 14617631

[B3] Brentano F. Schorr O. Ospelt C. Stanczyk J. Gay R. E. Gay S. (2007). Pre–B cell colony‐enhancing factor/visfatin, a new marker of inflammation in rheumatoid arthritis with proinflammatory and matrix‐degrading activities. Arthritis and Rheumatism 56 (9), 2829–2839. 10.1002/art.22833 17763446

[B4] Buket Basmanav F. Kose G. T. Hasirci V. (2008). Sequential growth factor delivery from complexed microspheres for bone tissue engineering. Biomaterials 29 (31), 4195–4204. 10.1016/j.biomaterials.2008.07.017 18691753

[B5] Camorani S. Hill B. S. Fontanella R. Greco A. Gramanzini M. Auletta L. (2017). Inhibition of bone marrow-derived mesenchymal stem cells homing towards triple-negative breast cancer microenvironment using an Anti-PDGFRbeta aptamer. Theranostics 7 (14), 3595–3607. 10.7150/thno.18974 28912898 PMC5596446

[B6] Caplan A. I. (2007). Adult mesenchymal stem cells for tissue engineering *versus* regenerative medicine. J. Cell. Physiology 213 (2), 341–347. 10.1002/jcp.21200 17620285

[B7] Charbonneau M. Lavoie R. R. Lauzier A. Harper K. McDonald P. P. Dubois C. M. (2016). Platelet-derived growth factor receptor activation promotes the prodestructive Invadosome-Forming phenotype of synoviocytes from patients with rheumatoid arthritis. J. Immunol. 196 (8), 3264–3275. 10.4049/jimmunol.1500502 26976956

[B8] Chen L. Li J. Tu S. Yang S. Lei Y. Wang L. (2025). Injectable hydrogel microspheres for osteoarthritis therapy *via* endogenous mesenchymal stem cells homing and chondrogenic differentiation enhancement. Adv. Healthc. Mater. 10.1002/adhm.202504727 41312903

[B9] Chung J. Grammer T. C. Lemon K. P. Kazlauskas A. Blenis J. (1994). PDGF- and insulin-dependent pp70S6k activation mediated by phosphatidylinositol-3-OH kinase. Nature 370 (6484), 71–75. 10.1038/370071a0 8015612

[B10] Correa D. Lietman S. A. (2017). Articular cartilage repair: current needs, methods and research directions. Semin. Cell Dev. Biol. 62, 67–77. 10.1016/j.semcdb.2016.07.013 27422331

[B11] Crisan M. Yap S. Casteilla L. Chen C. W. Corselli M. Park T. S. (2008). A perivascular origin for mesenchymal stem cells in multiple human organs. Cell Stem Cell 3 (3), 301–313. 10.1016/j.stem.2008.07.003 18786417

[B12] Cucchiarini M. Madry H. Ma C. Thurn T. Zurakowski D. Menger M. D. (2005). Improved tissue repair in articular cartilage defects *in vivo* by rAAV-Mediated overexpression of human fibroblast growth factor 2. Mol. Ther. 12 (2), 229–238. 10.1016/j.ymthe.2005.03.012 16043094

[B13] Depypere M. Morgenstern M. Kuehl R. Senneville E. Moriarty T. F. Obremskey W. T. (2020). Pathogenesis and management of fracture-related infection. Clin. Microbiol. Infect. 26 (5), 572–578. 10.1016/j.cmi.2019.08.006 31446152

[B14] Dhahri D. Sato-Kusubata K. Ohki-Koizumi M. Nishida C. Tashiro Y. Munakata S. (2016). Fibrinolytic crosstalk with endothelial cells expands murine mesenchymal stromal cells. Blood 128 (8), 1063–1075. 10.1182/blood-2015-10-673103 27283026

[B15] Dieppe P. A. Lohmander L. S. (2005). Pathogenesis and management of pain in osteoarthritis. Lancet 365 (9463), 965–973. 10.1016/S0140-6736(05)71086-2 15766999

[B16] Fortier L. A. Barker J. U. Strauss E. J. McCarrel T. M. Cole B. J. (2011). The role of growth factors in cartilage repair. Clin. Orthop. and Relat. Res. 469 (10), 2706–2715. 10.1007/s11999-011-1857-3 21403984 PMC3171543

[B17] Fredriksson L. Li H. Eriksson U. (2004). The PDGF family: four gene products form five dimeric isoforms. Cytokine and Growth Factor Rev. 15 (4), 197–204. 10.1016/j.cytogfr.2004.03.007 15207811

[B18] Goldring M. B. Otero M. (2011). Inflammation in osteoarthritis. Curr. Opin. Rheumatology 23 (5), 471–478. 10.1097/BOR.0b013e328349c2b1 21788902 PMC3937875

[B19] Hamilton J. L. Nagao M. Levine B. R. Chen D. Olsen B. R. Im H. J. (2016). Targeting VEGF and its receptors for the treatment of osteoarthritis and associated pain. J. Bone Mineral Res. 31 (5), 911–924. 10.1002/jbmr.2828 27163679 PMC4863467

[B20] Heinegård D. Saxne T. (2010). The role of the cartilage matrix osteoarthritis. Nat. Rev. Rheumatol. 7 (1), 50–56. 10.1038/nrrheum.2010.198 21119607

[B21] Heinegard D. Saxne T. (2011). The role of the cartilage matrix in osteoarthritis. Nat. Rev. Rheumatol. 7 (1), 50–56. 21119607 10.1038/nrrheum.2010.198

[B22] Heldin C. H. Lennartsson J. (2013). Structural and functional properties of platelet-derived growth factor and stem cell factor receptors. Cold Spring Harb. Perspect. Biol. 5 (8), a009100. 10.1101/cshperspect.a009100 23906712 PMC3721287

[B23] Huang K. Cai H. (2025). The role of chondrocyte senescence in osteoarthritis pathogenesis and therapeutic implications. Exp. Gerontol., 208. 10.1016/j.exger.2025.112828 40651670

[B24] Huebsch N. Lippens E. Lee K. Mehta M. Koshy S. T. Darnell M. C. (2015). Matrix elasticity of void-forming hydrogels controls transplanted-stem-cell-mediated bone formation. Nat. Mater. 14 (12), 1269–1277. 10.1038/nmat4407 26366848 PMC4654683

[B25] Hunziker E. B. (2002). Articular cartilage repair: basic science and clinical progress. A review of the current status and prospects. Osteoarthr. Cartil. 10 (6), 432–463. 10.1053/joca.2002.0801 12056848

[B26] Hye-Ryong Shim A. Liu H. Focia P. J. Chen X. Lin P. C. He X. (2010). Structures of a platelet-derived growth factor/propeptide complex and a platelet-derived growth factor/receptor complex. Proc. Natl. Acad. Sci. 107 (25), 11307–11312. 10.1073/pnas.1000806107 20534510 PMC2895058

[B27] Indrawattana N. Chen G. Tadokoro M. Shann L. H. Ohgushi H. Tateishi T. (2004). Growth factor combination for chondrogenic induction from human mesenchymal stem cell. Biochem. Biophysical Res. Commun. 320 (3), 914–919. 10.1016/j.bbrc.2004.06.029 15240135

[B28] Kratchmarova I. Blagoev B. Haack-Sorensen M. Kassem M. Mann M. (2005). Mechanism of divergent growth factor effects in mesenchymal stem cell differentiation. Science 308 (5727), 1472–1477. 10.1126/science.1107627 15933201

[B29] Lemmon M. A. Schlessinger J. (2010). Cell signaling by receptor tyrosine kinases. Cell 141 (7), 1117–1134. 10.1016/j.cell.2010.06.011 20602996 PMC2914105

[B30] Li X. Eriksson U. (2003). Novel PDGF family members: PDGF-C and PDGF-D. Cytokine Growth Factor Rev. 14 (2), 91–98. 10.1016/s1359-6101(02)00090-4 12651221

[B31] Li X. Gibson G. Kim J. S. Kroin J. Xu S. van Wijnen A. J. (2011). MicroRNA-146a is linked to pain-related pathophysiology of osteoarthritis. Gene 480 (1-2), 34–41. 10.1016/j.gene.2011.03.003 21397669 PMC3095758

[B32] Liebesny P. H. Byun S. Hung H. H. Pancoast J. R. Mroszczyk K. A. Young W. T. (2016). Growth factor-mediated migration of bone marrow progenitor cells for accelerated scaffold recruitment. Tissue Eng. Part A 22 (13-14), 917–927. 10.1089/ten.TEA.2015.0524 27268956 PMC4948211

[B33] Lories R. J. Luyten F. P. (2010). The bone–cartilage unit in osteoarthritis. Nat. Rev. Rheumatol. 7 (1), 43–49. 10.1038/nrrheum.2010.197 21135881

[B34] Lutolf M. P. Hubbell J. A. (2005). Synthetic biomaterials as instructive extracellular microenvironments for morphogenesis in tissue engineering. Nat. Biotechnol. 23 (1), 47–55. 10.1038/nbt1055 15637621

[B35] Morikawa S. Mabuchi Y. Kubota Y. Nagai Y. Niibe K. Hiratsu E. (2009). Prospective identification, isolation, and systemic transplantation of multipotent mesenchymal stem cells in murine bone marrow. J. Exp. Med. 206 (11), 2483–2496. 10.1084/jem.20091046 19841085 PMC2768869

[B36] Mourkioti F. Rosenthal N. (2005). IGF-1, inflammation and stem cells: interactions during muscle regeneration. Trends Immunol. 26 (10), 535–542. 10.1016/j.it.2005.08.002 16109502

[B37] Mueller M. B. Tuan R. S. (2008). Functional characterization of hypertrophy in chondrogenesis of human mesenchymal stem cells. Arthritis and Rheumatism 58 (5), 1377–1388. 10.1002/art.23370 18438858 PMC3612425

[B38] Murphy M. B. Moncivais K. Caplan A. I. (2013). Mesenchymal stem cells: environmentally responsive therapeutics for regenerative medicine. Exp. Mol. Med. 45 (11), e54. 10.1038/emm.2013.94 24232253 PMC3849579

[B39] Omata Y. Tachibana H. Aizaki Y. Mimura T. Sato K. (2023). Essentiality of Nfatc1 short isoform in osteoclast differentiation and its self-regulation. Sci. Rep. 13 (1), 18797. 10.1038/s41598-023-45909-3 37914750 PMC10620225

[B40] Park-Min K. H. (2019). Metabolic reprogramming in osteoclasts. Semin. Immunopathol. 41 (5), 565–572. 10.1007/s00281-019-00757-0 31552471 PMC7671717

[B41] Peng Y. Wu S. Li Y. Crane J. L. (2020). Type H blood vessels in bone modeling and remodeling. Theranostics 10 (1), 426–436. 10.7150/thno.34126 31903130 PMC6929606

[B42] Povysil C. Kana, R. Horak M. Kana M. (2025). Properties and functions of myochondrocytes and myochondroblasts in different human cartilage Tissues-An overview. Cells 14 (19). 10.3390/cells14191504 41090733 PMC12523445

[B43] Roskoski R. Jr. (2018). The role of small molecule platelet-derived growth factor receptor (PDGFR) inhibitors in the treatment of neoplastic disorders. Pharmacol. Res. 129, 65–83. 10.1016/j.phrs.2018.01.021 29408302

[B44] Sanchez-Lopez E. Coras R. Torres A. Lane N. E. Guma M. (2022). Synovial inflammation in osteoarthritis progression. Nat. Rev. Rheumatol. 18 (5), 258–275. 10.1038/s41584-022-00749-9 35165404 PMC9050956

[B45] Schmidt M. B. Chen E. H. Lynch S. E. (2006). A review of the effects of insulin-like growth factor and platelet derived growth factor on *in vivo* cartilage healing and repair. Osteoarthr. Cartil. 14 (5), 403–412. 10.1016/j.joca.2005.10.011 16413799

[B46] Schneider G. Montaseri A. Busch F. Mobasheri A. Buhrmann C. Aldinger C. (2011). IGF-1 and PDGF-bb suppress IL-1β-Induced cartilage degradation through down-regulation of NF-κB signaling: involvement of Src/PI-3K/AKT pathway. PLoS One 6 (12). 10.1371/journal.pone.0028663 22194879 PMC3237481

[B47] Shi S. Mercer S. Eckert G. J. Trippel S. B. (2009). Growth factor regulation of growth factors in articular chondrocytes. J. Biol. Chem. 284 (11), 6697–6704. 10.1074/jbc.M807859200 19136669 PMC2652312

[B48] Sokolove J. Lepus C. M. (2013). Role of inflammation in the pathogenesis of osteoarthritis: latest findings and interpretations. Ther. Adv. Musculoskelet. Dis. 5 (2), 77–94. 10.1177/1759720X12467868 23641259 PMC3638313

[B49] Su W. Liu G. Liu X. Zhou Y. Sun Q. Zhen G. (2020). Angiogenesis stimulated by elevated PDGF-BB in subchondral bone contributes to osteoarthritis development. JCI Insight 5 (8). 10.1172/jci.insight.135446 32208385 PMC7205438

[B50] Tallquist M. D. Soriano P. (2003). Cell autonomous requirement for PDGFRα in populations of cranial and cardiac neural crest cells. Development 130 (3), 507–518. 10.1242/dev.00241 12490557

[B51] Tuckermann J. Adams R. H. (2021). The endothelium-bone axis in development, homeostasis and bone and joint disease. Nat. Rev. Rheumatol. 17 (10), 608–620. 10.1038/s41584-021-00682-3 34480164 PMC7612477

[B52] Valius M. Kazlauskas A. (1993). Phospholipase C-gamma 1 and phosphatidylinositol 3 kinase are the downstream mediators of the PDGF receptor's mitogenic signal. Cell 73 (2), 321–334. 10.1016/0092-8674(93)90232-f 7682895

[B53] Wang J. Fang C. L. Noller K. Wei Z. Liu G. Shen K. (2023). Bone-derived PDGF-BB drives brain vascular calcification in male mice. J. Clin. Invest 133 (23), e168447. 10.1172/JCI168447 37815871 PMC10688993

[B54] Wang Y. Li Y. Jiang J. Hong Y. Gao S. Hua C. (2025a). Ferritinophagy in inflammatory and autoimmune diseases: mechanistic insights and therapeutic potentials. Autoimmun. Rev. 25, 103954. 10.1016/j.autrev.2025.103954 41176258

[B55] Wang Z. Zhu P. Li H. Cheng J. Cai Y. (2025b). PDGF-BB inhibits F-actin formation and chondrocyte dedifferentiation in osteoarthritis *via* oxygen-dependent HIF-1α/SCIN regulation and RhoA/ROCK signaling inhibition. Eur. J. Pharmacol. 1007. 10.1016/j.ejphar.2025.178280 41135736

[B56] Xiao J. I. N. Chen X. Xu L. Zhang Y. Yin Q. Wang F. (2014). PDGF regulates chondrocyte proliferation through activation of the GIT1-and PLCγ1-mediated ERK1/2 signaling pathway. Mol. Med. Rep. 10 (5), 2409–2414. 10.3892/mmr.2014.2506 25175053

[B57] Xu J. Wu H. F. Ang E. S. M. Yip K. Woloszyn M. Zheng M. H. (2009). NF-kappaB modulators in osteolytic bone diseases. Cytokine Growth Factor Rev. 20 (1), 7–17. 10.1016/j.cytogfr.2008.11.007 19046922

[B58] Zhao M. Perry J. M. Marshall H. Venkatraman A. Qian P. He X. C. (2014). Megakaryocytes maintain homeostatic quiescence and promote post-injury regeneration of hematopoietic stem cells. Nat. Med. 20 (11), 1321–1326. 10.1038/nm.3706 25326798

[B59] Zhao G.-Z. Zhang L. Q. Liu Y. Fang J. Li H. Z. Gao K. H. (2016). Effects of platelet-derived growth factor on chondrocyte proliferation, migration and apoptosis *via* regulation of GIT1 expression. Mol. Med. Rep. 14 (1), 897–903. 10.3892/mmr.2016.5291 27220359

[B60] Zhou L. Xu J. Schwab A. Tong W. Xu J. Zheng L. (2023). Engineered biochemical cues of regenerative biomaterials to enhance endogenous stem/progenitor cells (ESPCs)-mediated articular cartilage repair. Bioact. Mater 26, 490–512. 10.1016/j.bioactmat.2023.03.008 37304336 PMC10248882

[B61] Zhu P. Wang Z. Sun Z. Liao B. Cai Y. (2021). Recombinant platelet‐derived growth factor‐BB alleviates osteoarthritis in a rat model by decreasing chondrocyte apoptosis *in vitro* and *in vivo* . J. Cell. Mol. Med. 25 (15), 7472–7484. 10.1111/jcmm.16779 34250725 PMC8335691

[B62] Zimmer J. Chen L. Tredget E. E. Liu C. Wu Y. (2009). Analysis of allogenicity of mesenchymal stem cells in engraftment and wound healing in mice. PLoS One 4 (9). 10.1371/journal.pone.0007119 19771171 PMC2743192

